# Brain-derived neurotrophic factor, but not body weight, correlated with a reduction in depression scale scores in men with metabolic syndrome: a prospective weight-reduction study

**DOI:** 10.1186/1758-5996-6-18

**Published:** 2014-02-13

**Authors:** I-Te Lee, Chia-Po Fu, Wen-Jane Lee, Kae-Woei Liang, Shih-Yi Lin, Chu-Jen Wan, Wayne Huey-Herng Sheu

**Affiliations:** 1 Division of Endocrinology and Metabolism, Department of Internal Medicine, Taichung Veterans General Hospital, No. 1650, Section 4, Taiwan Boulevard, Taichung 40705, Taiwan; 2 School of Medicine, Chung Shan Medical University, Taichung, Taiwan; 3 School of Medicine, National Yang-Ming University, Taipei, Taiwan; 4 Department of Medical Research, Taichung Veterans General Hospital, Taichung, Taiwan; 5 Cardiovascular Center, Taichung Veterans General Hospital, Taichung, Taiwan; 6 Department of Nutrition, Hung-Kuang University, Taichung, Taiwan; 7 Institute of Medical Technology, National Chung-Hsing University, Taichung, Taiwan

**Keywords:** Exercise, Lifestyle intervention, Obesity, Zung self-rating depression scale

## Abstract

**Background:**

Obesity, a critical component of metabolic syndrome (MetS), is associated with depression. Deficiency of brain-derived neurotrophic factor (BDNF) is involved in the mechanism of depression. We hypothesized that weight reduction would improve depressive symptoms via increasing BDNF levels in obese men.

**Methods:**

Male adults with obesity were enrolled in a weight-reduction program for twelve weeks. All subjects underwent daily caloric restriction and an exercise program which was regularly assessed in group classes. Fasting blood samples and Zung Self-Rating Depression Scale (Zung SDS) scores were collected for assessments before and after the study.

**Results:**

A total of 36 subjects completed this program. The average reduction in body weight was 8.4 ± 5.1 kg (8.8 ± 5.1%, P < 0.001). Fasting serum BDNF significantly increased after the study (from 40.4 ± 7.8 to 46.9 ± 8.9 ng/ml, P < 0.001). However, the depression symptoms, as assessed by the Zung Self-Rating Depression Scale (Zung SDS), did not reduce significantly (P = 0.486). Divided into subgroups based on changes in BDNF, Zung SDS scores were significantly reduced in subjects with greater BDNF increase than in those with minor BDNF change (-3.9 ± 6.2 vs. 2.3 ± 6.7, P = 0.009). The increased percentage of BDNF was inversely correlated with the change in Zung SDS (*r* = -0.380, P = 0.022). Multivariate regression analysis showed that reduction in BDNF was independently associated with change in Zung SDS (95% confidence interval -0.315 to -0.052, P = 0.008).

**Conclusion:**

Zung SDS only significantly improved in men with increased fasting BDNF levels after a lifestyle intervention.

**Trial registration:**

(NCT01065753, ClinicalTrials.gov)

## Introduction

Metabolic syndrome (MetS), a cluster of risk factors for cardiovascular disease, is associated with several chronic diseases and has become an important public health concern due to its high prevalence [1-3]. Obesity is considered a critical target for treatment due to its pathophysiological role in MetS [4-6]. Weight reduction by dietary and exercise intervention can prevent several sequential complications of MetS [7-9].

Adult obesity is also associated with intellectual disabilities [10]. Decreased hippocampal volume and advanced brain atrophy have been reported in subjects with central obesity [11]. Furthermore, MetS could predict cognitive impairment in prospective studies [12,13]. MetS is clearly associated with dysfunction of the central nervous system; however, the underlying factors contributing to the pathogenesis of the central nervous system are not well understood. It was postulated that inflammation was associated with cognitive dysfunction in MetS [12-14]. Several studies have also suggested that some peripheral signals, crossing the blood brain barrier, may be involved in neuroprotective pathways [15-17].

Brain-derived neurotrophic factor (BDNF), an important neurotrophin in the development of neurons and the regulation of synaptic activities, exists not only in the central nervous system but also in the peripheral blood with a positive correlation [18-21]. The process of learning and memory formation are associated with the activation of tyrosine receptor kinase B (TrkB) through the binding of BDNF [22]. BDNF has been reported to be associated with depression, and the severity of depression might be affected by changes in BDNF after antidepressant treatment [23,24].

Furthermore, BDNF helps maintain the energy homeostasis that suppresses food intake and increases energy expenditure [25,26]. Circulating BDNF was reported to be increased in obese women, and high BDNF levels were observed in subjects with MetS [27-29]. Conversely, low BDNF concentrations were also observed in subjects with obesity, MetS or diabetes [30,31]. Martin et al. [32] reported improved cognitive function and increased circulating BDNF in rats undergoing energy restriction. To examine the hypothesis that an increase in circulating BDNF concentration can improve central nervous symptoms in men during weight reduction, a prospective study was conducted to assess BDNF and depression score in men with MetS after a lifestyle intervention.

## Methods

### Subjects

This study was conducted in the Division of Endocrinology and Metabolism at Taichung Veterans General Hospital. The inclusion criteria were men with MetS as defined by the International Diabetes Federation (IDF), who fulfilled the following conditions: central obesity with a waist circumference greater than 90 cm, plus two or more of the following four components: (1) triglyceride equal to or greater than 150 mg/dL (1.7 mmol/L), (2) high-density lipoprotein (HDL) cholesterol less than 40 mg/dL (1.0 mmol/L), (3) blood pressure equal to or greater than 130/85 mmHg or using antihypertensive medications and (4) fasting glucose equal to or greater than 100 mg/dL (5.6 mmol/L) [4]. Exclusion criteria were: (1) aged under 20 or over 75 years, (2) history of diabetes or taking anti-diabetic medications, (3) history of psychological disorders or medications for schizophrenia or bipolar disorder, (4) endocrine diseases such as thyroid or adrenal disorders, (5) acute or chronic renal diseases with serum creatinine levels greater than 200 mmol/L, (6) alanine aminotransferase or aspartate aminotransferase levels three times greater than the normal upper limit, or liver cirrhosis, (7) acute or chronic infectious diseases, (8) severe systemic diseases such as malignant or immune disorders, (9) addiction to alcohol or drugs, (10) taking any medication which changes body weight, such as steroid, (11) changes in medications for hypertension, hyperlipidemia, anti-platelet or anti-inflammation in the past month, (12) limitations to following the regular dietary and exercise intervention based on clinical judgment or the participant’s personal reasons.

### Procedure

All the enrolled subjects took part in a scheduled twelve-week weight-reduction program. Each subject received instructions from a registered dietitian on how to maintain a 1200 Kcal/day diet, divided into 300 Kcal at breakfast, 400 Kcal at lunch and 500 Kcal at dinner. Subjects were required to attend eight classes of group training in lifestyle change. The content of each three-hour class included: (1) assessment of body weight and diet diaries by the dietitian, (2) an educational program for diet control, exercise promotion and target setting, (3) a discussion among the participants, the dietitian and doctors specializing in cardiology or endocrinology, and (4) a 50-minute practice session with moderate aerobic exercise under the supervision of trained instructors and with the attendance of doctors. A reduction of 10% in basal body weight was planned and no medication changes were allowed during the study. The study was approved by the Institutional Review Board of Taichung Veterans General Hospital and written informed consent was provided by all participants (NCT number: 01065753).

### Measurements

Overnight fasting blood samples were collected at baseline and at the end of the study for measurements of glucose, insulin, lipids, liver enzymes, creatinine, C-reactive protein (CRP) and BDNF. Study subjects were assessed using the Zung Self-Rating Depression Scale (Zung SDS). There were 20 items on the depression questionnaire, either positive or negative, which the study subjects were required to grade on a scale of 1 to 4. The severity score was calculated by formula conversion [33].

Glucose, creatinine, triglyceride, cholesterol and liver enzymes were measured by commercial kits (Beckman Coulter, Fullerton, USA). Insulin, HDL cholesterol and low-density lipoprotein (LDL) cholesterol levels were also measured by commercial kits (Roche Diagnostics GmbH, Mannheim, Germany). For quantitative evaluation of insulin resistance, the homeostasis model assessment of insulin resistance (HOMA-IR) index was calculated using the following equation: fasting insulin (μIU/mL)*fasting glucose (mmol/L)/22.5 [34]. CRP was measured by the immunochemical assay of purified Duck IgY (ΔFc) antibodies (Good Biotech Corp., Taichung, Taiwan); the mean intra-assay and inter-assay CVs for CRP were 1.4% and 1.4%, with an analytical sensitivity of 0.1 mg/L. Human BDNF was measured by a commercially available immunoassay kit (R & D Systems, Minneapolis, USA), and the mean intra-assay and inter-assay CVs for BDNF were 4.1% and 9.0%, respectively, with an analytical sensitivity of 0.02 ng/mL. There was no significant cross-reactivity or interference with nerve growth factor, neurotrophin-3 and neurotrophin-4 prepared at a concentration of 50 ng/mL in a BDNF control.

### Statistics

All descriptive data were presented as mean ± standard deviation (SD). Statistical analyses were conducted with the non-parametric Wilcoxon Signed Rank test to compare the difference before and after intervention. The relationship between the change in Zung SDS and the associated risk factors was determined by Spearman’s correlation. A Mann-Whitney test was used to detect the difference between the two groups. Multivariate linear regression analysis was employed to analyze the factors associated with the change in Zung SDS. Statistical analyses were performed using SPSS 12.0 (Chicago, IL, USA).

## Results

**Figure 1:**
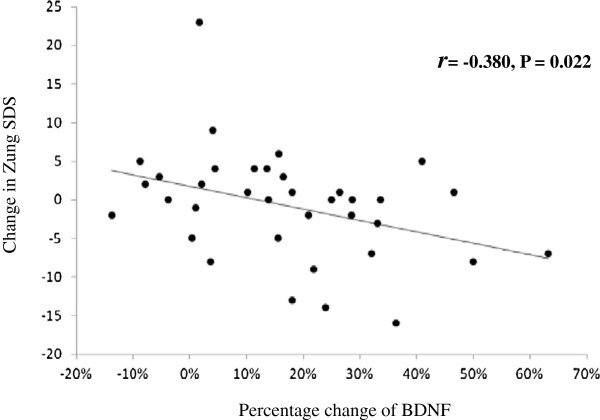
**Relationship between the percentage changes in BDNF and changes in the Zung Self-Rating Depression Scale (Zung SDS) score; *r*** **= -0.380, *P*** **= 0.022.**

Of the 40 subjects with MetS enrolled for the study, 36 subjects completed the weight-reduction program. The average weight was significantly reduced by 8.4 ± 5.1 kg (8.7 ± 5.1%, P < 0.001). The average serum concentration of BDNF was significantly increased at the end of the study (from 40.4 ± 7.8 to 46.9 ± 8.9 ng/ml, P < 0.001). However, the Zung SDS did not significantly differ from the baseline by the end of the study (from 37.6 ± 8.8 to 36.9 ± 7.2, P = 0.486). To determine the effect of BDNF, all subjects were equally divided into two groups based on the percentage change in BDNF (median of 16.1%) (Table 1). The baseline fasting insulin (P = 0.034) and HOMA-IR (P = 0.029) were higher in subjects with a greater BDNF increase than in those with a minor BDNF change. Table 2 shows the characteristics of both groups before and after the study. No significant differences were observed in change of body weight, adipokines, CRP or any of the MetS components between these two groups at the end of the study. Interestingly, significant reduction in Zung SDS in subjects with a greater BDNF increase was found compared to those with a minor BDNF change (-3.9 ± 6.2 vs. 2.3 ± 6.7, P = 0.008). Figure 1 shows a significant correlation between the changes in BDNF and in Zung SDS (*r* = -0.380, P = 0.022). However, change in body weight or MetS components were not correlated with Zung SDS (Table 3). Based on the regression model, the increase in BDNF was an independent factor for the reduction of Zung SDS, after adjusting for age, insulin resistance, and changes in weight. (Table 4). Furthermore, baseline BDNF seemed to be higher in the minor BDNF change group than in the other. However, after adjusting for the baseline BDNF levels, the change in BDNF was still associated with the reduction in Zung SDS (95% confidence interval between -0.355 and 0.073, P = 0.004).

**Table 1:** **The baseline data of subjects based on percentage change in BDNF**^**1**^

	**Minor BDNF change**	**Greater BDNF increase**	**P**
	**(n = 18)**	**(n = 18)**	
Age (year)	41±9	47±11	0.084
Body weight (kg)	95.6±13.8	98.3±11.4	0.319
BMI (kg/m^2^)	33.5±4.2	33.4±3.6	0.849
Waist circumference (cm)	108.1±10.4	110.0±7.8	0.288
Systolic BP (mmHg)	129±16	141±18	0.059
Diastolic BP (mmHg)	79±12	82±13	0.438
Fasting glucose (mmol/L)	5.5±0.9	5.7±0.7	0.090
Fasting insulin (μIU/ml)	15.6±5.2	22.8±14.0	0.034
HOMA IR	3.9±1.5	5.9±3.7	0.029
Triglyceride (mmol/L)	4.0±7.1	2.5±1.1	0.812
Total cholesterol (mmol/L)	5.5±1.6	5.0±0.7	0.393
LDL cholesterol (mmol/L)	3.3±0.9	3.2±0.6	0.486
HDL cholesterol (mmol/L)	1.0±0.2	1.0±0.2	0.568
Serum creatinine	81.4±12.9	81.2±16.9	0.887
AST (U/L)	28.7±10.4	34.4±11.4	0.081
ALT (U/L)	40±17	48±21	0.241
CRP (mg/L)	3.5±4.8	2.2±2.0	0.558
BDNF (ng/ml)	43.7±7.5	37.2±6.8	0.011
Zung SDS	37.3±7.1	38.0±10.4	0.646

^**1**^cutoff value: median (16.1%) of the change percentage of BDNF.

ALT = alanine aminotransferase, AST = aspartate aminotransferase, BDNF = brain-derived neurotrophic factor, BMI = body mass index, BP = blood pressure, CRP = C-reactive protein, HDL = high-density lipoprotein, HOMA-IR = homeostasis model assessment of insulin resistance, LDL = low-density lipoprotein, Zung SDS = Zung Self-Rating Depression Scale.

**Table 2:** **Alteration in clinical data of subjects grouped based on percentage change in BDNF**^**1**^

	**Minor BDNF change**	**Greater BDNF increase**	**P**
	**(n = 18)**	**(n = 18)**	
Body weight (kg)	-7.7±4.8*	-9.1±5.4*	
(%)	-8.2%±5.2%	-9.2%±5.2%	0.752
Waist circumference (cm)	-9.3±5.9*	-10.0±5.8*	
(%)	-8.5%±5.1%	-9.0%±5.3%	0.812
Systolic BP (mmHg)	-10±18*	-20±22*	
(%)	-6.6%±12.9%	-13.6%±13.7%	0.129
Diastolic BP (mmHg)	-5±8*	-10±12*	
(%)	-6.4%±9.1%	-11.1%±14.1%	0.223
Fasting glucose (mmol/L)	-0.2±0.5	-0.4±0.6*	
(%)	-2.2%±8.7%	-5.9%±10.9%	0.159
HOMA IR	-1.0±1.0*	-2.3±3.3*	
(%)	-24.4%±19.6%	-31.9%±32.1%	0.229
Triglyceride (mmol/L)	-1.5± 3.2*	-0.8±0.9*	
(%)	-32.3%±21.5%	-30.9%±28.1%	0.752
Total cholesterol (mmol/L)	-0.6±0.9*	-0.1±0.6*	
(%)	-8.3%±13.5%	-3.0%±11.2%	0.164
LDL cholesterol (mmol/L)	-0.4±0.5*	-0.1±0.6	
(%)	-8.5%±18.0%	-2.1%±18.2%	0.206
HDL cholesterol (mmol/L)	0.1±0.1	0.1±0.3	
(%)	8.8%±15.2%	8.4%±25.0%	0.899
Serum creatinine	-4.5±8.2*	1.6±17.7	
(%)	-5.2%±9.2%	2.2%±18.0%	0.056
AST (U/L)	-7±10*	-11±10*	
(%)	-17.0%±25.0%	-28.1%±24.1%	0.129
ALT (U/L)	-12±14*	-19±13*	
(%)	-25.6%±27.6%	-36.8%±19.6%	0.248
CRP (mg/L)	-1.7±4.7*	-0.7±2.1	
(%)	-15.9%±78.1%	-1.6%±60.3%	0.343
BDNF (ng/ml)	0.7±1.9	5.7±2.2*	
(%)	3.2%±8.8%	31.3%±12.4%	<0.001
Zung SDS	2.3±6.7	-3.9±6.2*	
(%)	8.8%±23.9%	-7.8%±13.9%	0.009

*means P value less than 0.05 intra group.

^**1**^ cutoff value: median (16.1%) of the change percentage of BDNF.

ALT = alanine aminotransferase, AST = aspartate aminotransferase, BDNF = brain-derived neurotrophic factor, BP = blood pressure, CRP = C-reactive protein, HDL = high-density lipoprotein, HOMA-IR = homeostasis model assessment of insulin resistance, LDL = low-density lipoprotein, Pc = post-challenge, Zung SDS = Zung Self-Rating Depression Scale.

**Table 3:** Correlations between change in Zung SDS and percentage change based on different variables after body weight reduction

	***r***	**P**
Body weight (%)	0.14	0.414
Waist circumference (%)	0.15	0.382
Systolic BP (%)	0.19	0.261
Diastolic BP (%)	0.19	0.279
Fasting glucose (%)	-0.11	0.540
Fasting insulin (%)	0.09	0.599
HOMA IR (%)	0.04	0.810
Total cholesterol (%)	-0.26	0.128
Triglyceride (%)	0.06	0.736
HDL cholesterol (%)	-0.19	0.267
LDL cholesterol (%)	0.19	0.270
Serum creatinine (%)	-0.20	0.232
AST (%)	0.02	0.907
ALT (%)	0.08	0.641
CRP (%)	-0.09	0.621

ALT = alanine aminotransferase, AST = aspartate aminotransferase, BDNF = brain-derived neurotrophic factor, BP = blood pressure, CRP = C-reactive protein, HDL = high-density lipoprotein, HOMA-IR = homeostasis model assessment of insulin resistance, LDL = low-density lipoprotein, Zung SDS = Zung Self-Rating Depression Scale.

**Table 4:** Effects of risk factors on the changes in Zung Self-Rating Depression Scale during the study

	**β**^**1**^	**B**^**2**^	**95% CI**		**P**
Baseline					
Age (years)	0.154	0.102	-0.115	0.319	0.346
HOMA-IR	0.249	0.592	-0.167	1.350	0.122
Change					
Body weight (%)	0.209	0.286	-0.155	0.728	0.196
BDNF (%)	-0.461	-0.184	-0.315	-0.052	0.008

^1^β = standardized coefficient, ^2^B = linear regression coefficient, BDNF = brain-derived neurotrophic factor, CRP = C-reactive protein, HOMA-IR = homeostasis model assessment of insulin resistance.

## Discussion

In the present study, lifestyle intervention by diet control and exercise promotion resulted in a significant body-weight reduction in men with MetS. BDNF was significantly increased after the weight-reduction program. However, the mean severity of depressive symptoms assessed by the Zung SDS did not significantly improve. An important finding of the present study was an inverse correlation between the changes in Zung SDS and circulating BDNF level. Our results showed the important role played by BDNF in improving depressive symptoms in men with MetS during the weight-reduction process. Nevertheless, the correlation between the reduction in body weight and change in BDNF was not significant in the present study. In fact, serum BDNF was not associated with fat components of the body [35]. Exercise training without significant body-weight reduction can also increase the circulating BDNF concentration [36,37]. It has been reported recently that only muscle-strengthening, but not aerobic exercise, could increase circulating BDNF levels in women [38]. However, the response of BDNF to exercise might differ between genders [39], and circulating BDNF levels might be greater after aerobic exercise, rather than muscle strengthening, in men [40]. Although serum BDNF change might be correlated with weight-reduction magnitude in subjects being treated for schizophrenia [41], the relationship between BDNF and body-weight might be different due to the influence of gender and antipsychotic treatment [32,42]. It has been postulated that the main response of BDNF to exercise originates in the central nervous system rather than adipose tissue [43,44]. Therefore, based on our findings, the alteration in BDNF might not be directly related to the magnitude of weight reduction in obese men. It has also been reported that only weight reduction was not capable of improving depressive symptoms [45].

The proportion of MetS was reported to be significantly higher in Asian men with depressive symptoms assessed by Zung SDS than in those without [46]. However, depressive severity might not be significantly altered with a 5% reduction in body weight, and may worsen with an advanced body-weight reduction of 10% [47]. It was also hypothesized that depression at baseline might hinder the effect of lifestyle intervention on weight reduction [48]. However, this hypothesis was not supported in a previous study on female subjects [49]. Recent data from the Look Action for Health in Diabetes (Look AHEAD) study, a large-scale study on lifestyle intervention, reported that baseline depressive symptoms did not attenuate the magnitude of weight reduction in subjects with Type 2 diabetes [50]. In the present study, neither the baseline condition nor the change in Zung SDS was correlated with the change in body weight. Our findings suggest that depressive symptoms do not attenuate the effect of lifestyle intervention, with a mean weight reduction of 8.7% in men with MetS.

Among the baseline characteristics assessed in the present study, only fasting insulin and insulin resistance (presented by HOMA-IR) were significantly associated with increase in BDNF. According to previous cross-sectional studies, insulin sensitivity was associated with circulating BDNF [30,51], and hyperinsulinemia was a risk factor for depression [15,52]. Although HOMA-IR was significantly improved at the end of the present study, the alteration in HOMA-IR was not significantly different between the group with a greater BDNF increase and the other. Neither fasting insulin nor HOMA-IR was significantly correlated with a reduction in Zung SDS after lifestyle intervention. The reduction in circulating insulin did not appear to appreciably improve depressive symptoms even with functional receptors expressed in the central nervous system [53]. Based on the other baseline parameters, the alterations in BDNF and Zung SDS were not significantly different between the subjects with higher fasting glucose (≥ 5.6 mmol/L) and those with normal fasting glucose (< 5.6 mmol/L) in the present study. Furthermore, the alterations in BDNF and Zung SDS were not significantly different between the subjects with higher (≥ median of 36.5) and lower (< 36.5) baseline Zung SDS. The alterations in Zung SDS and body weight were not significantly different between the subjects with higher (≥ median of 40.7 ng/ml) and lower one (BDNF < 40.7 ng/ml) baseline BDNF. In addition, our results showed that Zung SDS was not significantly correlated with inflammation.

Our study shows that the increase in BDNF was inversely correlated with the severity of depressive symptoms in men without psychological disorders. Despite the confounding effect from the open-label questionnaire answers, the study participants and the investigators were blinded to the BDNF data during the study. This finding is noteworthy because BDNF was found to be involved in the pathogenic mechanism of depression [44,54]. However, the motivation of enrolled subjects in a short-term study is usually expected to be stronger, and the effects of body-weight reduction may be greater than those in a long-term follow-up. In the Look AHEAD study, an astonishing reduction in cardiovascular risks was observed within the first year, but the benefits were gradually attenuated in the following years [55,56]. With a median follow-up of approximately 10 years, cardiovascular events were not significantly reduced in the Look AHEAD study [57]. Therefore, further study on the long-term effects of lifestyle intervention on BDNF and depression are warranted.

There were several limitations in the present study. First, the postprandial change in BDNF by meal test was not assessed. An increase in free fatty acid was recently reported to suppress circulating BNDF after a high-fat intake [58]. However, it was unclear whether the response of BDNF to fat intake was associated with the depressive symptoms after body weight reduction. Second, our findings should only be applied to men. It has recently been reported that the relationship between circulating BDNF level and body weight differs between men and women [59]. There seemed to be a gender difference in the response of BDNF and depression to body-weight reduction [32]. Third, the men with increased BDNF showed a significant decrease in Zung SDS. The assessment of BDNF appeared to demonstrate that lifestyle intervention had an effect on depression. However, based on the present data, it is unclear whether a BDNF supplement would ameliorate depressive symptoms or affect body weight. In addition, cognitive functions or other psychological disorders were not assessed in the present study. In consideration of the pleiotropic effects of BDNF [60,61], it is possible that there were more psychological benefits for those subjects with increased BDNF after lifestyle intervention. It has been reported that low BDNF could predict high mortality in women [62], but its predictive ability in men requires further long-term studies.

In conclusion, after a 12-week weight reduction program, a decrease in body weight increased serum BDNF in obese men with MetS. However, depressive symptoms, as assessed by Zung SDS, were only significantly improved in subjects with increased serum BDNF levels after lifestyle intervention, independent of changes in body weight.

## Abbreviations

ALT: Alanine aminotransferase; AST: Aspartate aminotransferase; BDNF: Brain-derived neurotrophic factor; BMI: Body mass index; CRP: C-reactive protein; HDL: High-density lipoprotein; HOMA-IR: Homeostasis model assessment of insulin resistance; IDF: International Diabetes Federation; LDL: Low-density lipoprotein; MetS: Metabolic syndrome; SD: Standard deviation; Zung SDS: Zung Self-Rating Depression Scale.

## Competing interests

The authors declare that they have no competing interests.

## Authors’ contributions

I-TL conducted the study, interpreted the data and wrote the manuscript; C-PF conducted the study; W-JL conducted the study, performed the data collection and analysis; K-WL, S-YL, C-JW conducted the study; and WH-HS conducted the study, interpreted the data and wrote the manuscript. All authors read and approved the final manuscript.
